# Editorial: Exploring the molecular mechanisms that regulate macrophage polarization

**DOI:** 10.3389/fimmu.2025.1599215

**Published:** 2025-04-16

**Authors:** David E. Nelson, Michal A. Olszewski

**Affiliations:** ^1^ Department of Biology, Middle Tennessee State University, Murfreesboro, TN, United States; ^2^ Department of Veterans’ Affairs, Ann Arbor Health System, Ann Arbor, MI, United States; ^3^ Division of Pulmonary & Critical Care Medicine, University of Michigan Medical School, Ann Arbor, MI, United States; ^4^ Department of Microbiology & Immunology, University of Michigan Medical School, Ann Arbor, MI, United States

**Keywords:** macrophage, macrophage polarization, innate immunity, inflammation, macrophage activation

Macrophages are ubiquitous innate immune cells found in almost every organ of the body. This family of phagocytes, represented by a variety of different subtypes, performs tissue-specific functions as diverse as maintaining surfactant homeostasis performed by alveolar macrophages in the lung (1) or synaptic pruning carried out by the brain’s microglial cells (2). However, these cells are united by a common role in host defense, providing a rapid response to microbial invaders and/or tissue damage. As part of this function, macrophages adopt activation states, also referred to as polarization, during different phases of an infection, cycling between a resting state (M0) in the absence of infection to a highly proinflammatory and antimicrobial state upon exposure to microbial ligands or inflammatory cytokines, referred to as M1. In contrast to this classical inflammatory activation, alternative macrophage activation states are referred to as M2, which can promote the resolution of inflammation and tissue repair, among other functions (3).

This M1/M2 paradigm is not without its detractors. Several alternative models and naming systems have been proposed over the years, and with good reason (4–8). At least 3 distinct types of M2 activation have been proposed using idealized *in vitro* condition systems (9), and these systems cannot fully capture the complexity of these phenotypic shifts, which are a composite of multiple stimuli in specific tissue microenvironments overlaid with their temporal fluctuations. Furthermore, the M1/M2 system may be inappropriate for describing the behavior of certain macrophage subtypes. For example, alveolar macrophages do not express or only minimally express many of the canonical M1 markers (10, 11) and, due to their plasticity, often display intermediate M1/M2 phenotypes (12). For all its flaws, the M1/M2 system persists as a utilitarian shorthand that remains helpful when describing the pro-inflammatory and anti-inflammatory extremes of the polarization continuum and the shifts of macrophage populations toward these extremes as part of specific disease processes (13–15).

Given the broad transcriptional reprogramming necessary for macrophages to transition from one polarization state to another, which is generally accepted to involve the altered expression of over 1,000 genes (16), significant attention has been focused on the core transcriptional regulators responsible for its control. The M1 state, as induced by interferon-gamma (IFNγ) and microbial ligands signaling via toll-like receptors, is largely controlled by STAT1 (17, 18), IRF1 (19, 20), and NF-κB (21), whereas the M2 state, as induced by interleukin (IL)-4, IL-10, and IL-13, is mainly controlled by STAT3, 6 (22), and PPARγ (23). These circuits are not separate but linked by a range of feedback loops and cross-inhibitory mechanisms that generally provide coherent shifts from one state to the other. Superimposed on these mechanisms are the activities of other transcription factors, including c-Myc (24), KLF4 (25), p53 (26), and HIF1 (27), which are better known for regulating other cellular processes, such as cell proliferation, apoptosis, and the response to hypoxia, but make significant contributions to the regulation of either or both the M1 and M2 states.

In this Research Topic, which includes 6 (Mini) Reviews and Original Research Articles, we explore some of the lesser-known and often surprising contributors to macrophage polarization. These include extrinsic factors of the macrophage microenvironment, such as the biomechanical properties of the extracellular matrix and non-cytokine signaling agents, as well as intrinsic factors ranging from transcriptional co-regulators that modulate the activity of core M1 and M2 transcription factors to long non-coding RNAs (lncRNAs) and microRNAs (miRNAs) that influence the process at a post-transcriptional level.

In the first set of articles exploring the role of the tissue microenvironment on macrophage polarization, Joshi et al. provided an Original Research article on how changes in tissue compliance or stiffness and integrin signaling contribute to this process. This work was motivated by the observation that mechanical properties differ markedly between tissues, with stiffness generally increasing in the disease states (e.g., infection, cancer, and fibrosis), which has the potential to impact macrophage phenotype through mechanotransduction. This was explored using an *in vitro* model system employing bone marrow-derived macrophages incubated on collagen-coated gels of varying stiffness and in parallel experiments with leukadherin-1 (LA1), a CD11b agonist, used to activate integrin-mediated mechanical signaling independent of the substrate. Here, the authors showed that softer substrates favor a macrophage host-defense phenotype. They also found that LA1 attenuates pro-inflammatory signaling by inhibiting NLRP3 activation. Given that a recent clinical trial using LA1 was halted as the drug was found to have no benefit to cancer patients, this work raises the prospect that LA1 could be repurposed as an immunomodulatory drug to treat select inflammatory diseases.

The next Original Research article featured in our topic highlights a novel interaction of macrophages with a specific microbial component. In this rigorous study with mouse bone marrow-derived, peritoneal, and tumor-associated macrophages (TAMs), Zhang et al. demonstrated the effect of the *Escherichia coli* adhesion portion of type I fimbriae (FimH), which drives macrophage polarization status. The FimH, via a TLR4-mediated mechanism, not only M1-polarized resting macrophages but also M1-reprogrammed M2-macrophages induced by IL-4 and IL-13 cytokines or a tumor microenvironment. These properties of FimH were then validated in human macrophages and were shown to have therapeutic relevance. FimH could enhance anti-cancer immunity in C57BL/6 mice implanted with B16F10 melanoma treated with an anti-PD-L1 antibody. These therapeutic effects were linked to the induction of M1 polarization in TAMs, a property that could be clinically explored as a future adjunct therapy to the PD-L1 antibody treatment of tumors.

Two review articles published in our Research Topic focused on the lesser-known effects of growth factors and their receptors on macrophage polarization status. The first article by Shen et al. reviewed fibroblast growth factor (FGF) signaling in macrophage polarization. The authors discussed the importance of the FGF/FGF receptor (FGFR) axis during homeostasis and disease processes. FGFs have diverse regulatory functions in physiological processes, promoting the growth and development of bones and organs. However, they also contribute to the development of diseases such as cancer and metastasis, inflammatory processes, and metabolic disorders. The relationship between macrophages and different FGFs is complex. For example, FGF1 and 2 promote M1-type activation, while FGF20 has been reported to inhibit these pathways. In contrast, low molecular weight variants of FGF2 and FGF7 are either directly or indirectly linked to the promotion of M2 pathways. Similarly, the M1/M2 macrophage polarization cues induce different subsets of FGFR expression by macrophages and the production of specific FGFs such as FGF23. Since both FGFs and macrophages are involved in the progression of tumors, autoimmune, and degenerative diseases, these interactions need to be further examined in their specific biological contexts.

In the second review, Kannan and Rutkowski discussed the effects of vascular endothelial growth factor receptor-3 (VEGFR-3), which is highly expressed in lymphatic endothelial cells. However, monocyte-derived cells also express VEGFR-3 in specific organs such as the lung and the gut, but also in tumors and other chronic diseases. For instance, VEGFR-3-expressing macrophages are protected from the undesirable activation of pyroptosis pathways in the context of microbial infection, which results in better clearance and less inflammatory collateral damage of the infected tissues. However, the effects of VGFR-3 signaling on macrophage polarization appear to be contextual, especially in chronic diseases.

Finally, a set of articles in our Research Topic focused on intrinsic modulators of the polarization transcriptional program. Here, we featured a mini-review by Wiggins et al., in which the authors covered the recent literature on the CBP/p300-interacting transactivator with the glutamic acid/aspartic acid-rich carboxy-terminal domain (CITED) family of transcriptional co-regulators. Here, they summarized recent literature to argue that CITED1 and 2 function as general controllers of the M1 transcriptional program by regulating access to CBP/p300. This histone acetyltransferase can be considered a convergence point for signaling pathways regulating M1-associated gene expression as STAT, IRF, NF-κB, and HIF1 proteins utilize it as a coactivator. Here, CITED2 operates as a co-repressor, directly competing with these transcription factors for binding to a common interaction surface in CBP/p300, thereby attenuating pro-inflammatory gene expression. Conversely, CITED1 appears to enhance the expression of select pro-inflammatory genes, although the precise mechanisms for this remain enigmatic.

The intrinsic regulation of macrophage status is exemplified by the unique function of a nucleic acid regulator, a lncRNA identified in metastatic cancer macrophages, which has been described in an Original Research article from Ahmad et al.. Here, the authors explored the role of the metastasis-associated lung adenocarcinoma 1 (MALAT1) lncRNA, which is upregulated when monocytes are differentiated into M2 macrophages (28). This study found that MALAT1 functions as an antagonist of microRNAs from the miR-30 family, including miR-30b, which is known to support M2-associated gene expression. In this way, MALAT1 was found to bias macrophage polarization toward the M1 state by suppressing M2 gene expression.

In conclusion, this collection of research and review articles describes recently discovered and lesser-known regulators of macrophage behavior and polarization. These are diverse in nature and operate in a variety of different mechanisms, including mechanotransduction, and transcriptional and post-transcriptional control, with some playing important roles in tumor macrophage behavior, leading to cancer suppression or promotion, while others are involved in the response to infection and acute tissue injury or play roles in autoimmune or chronic inflammatory diseases. The number of genes and pathways found to be involved in the macrophage polarization process continues to grow, further increasing the number of defined or hypothetical interactions in these regulatory networks. This apparent complexity speaks to the incredible sophistication of macrophage polarization and the need for its tight regulation. Our Research Topic highlights only a small number of these regulatory mechanisms, and future studies will build on these, helping us better understand their contributions to health and disease.
